# Phenotyping chronic tinnitus patients using self-report questionnaire data: cluster analysis and visual comparison

**DOI:** 10.1038/s41598-020-73402-8

**Published:** 2020-10-02

**Authors:** Uli Niemann, Petra Brueggemann, Benjamin Boecking, Wilhelm Mebus, Matthias Rose, Myra Spiliopoulou, Birgit Mazurek

**Affiliations:** 1 Faculty of Computer Science, Otto von Guericke University Magdeburg, Universitätsplatz 2, 39106 Magdeburg, Germany; 2 Tinnitus Center, Charité Universitaetsmedizin Berlin, Charitéplatz 1, 10117 Berlin, Germany; 3 Division of Psychosomatic Medicine, Medical Department, Charité Universitaetsmedizin Berlin, Hindenburgdamm 30, 12200 Berlin, Germany

**Keywords:** Computer science, Signs and symptoms

## Abstract

Chronic tinnitus is a complex, multi-factorial symptom that requires careful assessment and management. Evidence-based therapeutic approaches involve audiological and psychological treatment components. However, not everyone benefits from treatment. The identification and characterisation of patient subgroups (or “phenotypes”) may provide clinically relevant information. Due to the large number of assessment tools, data-driven methods appear to be promising. The acceptance of these empirical results can be further strengthened by a comprehensive visualisation. In this study, we used cluster analysis to identify distinct tinnitus phenotypes based on self-report questionnaire data and implemented a visualisation tool to explore phenotype idiosyncrasies. 1228 patients with chronic tinnitus from the Charité Tinnitus Center in Berlin were included. At baseline, each participant completed 14 questionnaires measuring tinnitus distress, -loudness, frequency and location, depressivity, perceived stress, quality of life, physical and mental health, pain perception, somatic symptom expression and coping attitudes. Four distinct patient phenotypes emerged from clustering: *avoidant group* (56.8%), *psychosomatic group (14.1%)*, *somatic group* (15.2%), and *distress group* (13.9%). Radial bar- and line charts allowed for visual inspection and juxtaposition of major phenotype characteristics. The phenotypes differed in terms of clinical information including psychological symptoms, quality of life, coping attitudes, stress, tinnitus-related distress and pain, as well as socio-demographics. Our findings suggest that identifiable patient subgroups and their visualisation may allow for stratified treatment strategies and research designs.

## Introduction

Tinnitus, the perception of a phantom sound in absence of an external sound source, is a complex multi-factorially caused and maintained phenomenon. It is estimated to affect 10% and 15% of the adult population^1^. The associated annual economic burden amounts to US$19.4 billion in the United States^2^, and €6.8 billion in the Netherlands alone^3^. Clinical assessment of tinnitus is challenging due to its various heterogeneities. Tinnitus patients can differ with respect to perception of tinnitus (laterality, pitch, sound characteristics, frequency, permanence, chronicity), risk factors (including hearing loss, temporomandibular joint disorder, aging), comorbidities (including hyperacusis, depression, sleep disorders), perceived distress, and treatment responses^4^. These differences make the identification of a suitable treatment difficult. Currently, there is no consensus on or gold standard for a therapy form that is effective for every patient. Sound therapy (masking) alone is not sufficient to significantly improve tinnitus loudness and severity^5^. Informational counselling (minimal contact education) was found to be effective for subgroups of patients^6,7^. While some studies affirm the efficacy of cognitive behavioural therapy (CBT) in reducing tinnitus impairment and distress^8^, others conclude that CBT is not superior to other treatments or no treatment in improving subjective tinnitus loudness and quality of life^9^. Similarly, tinnitus retraining (TRT) was found to be helpful in reducing tinnitus impairment and quality of life in some studies^10,11^, but not others^12^. Due to the heterogeneous nature of the tinnitus symptom as well as the unclear evidence-base as to its treatment and management, the identification of patient subgroups is vital to stratify individual pathophysiology and treatment pathways^13–15^.

Since clinically relevant subgroups have not been established yet, clustering emerges as a promising approach to identify distinct tinnitus *phenotypes* in a data-driven, hypothesis-free way. Clustering is the process of grouping subjects into multiple groups or clusters. The goal is that subjects within a group are similar to another and dissimilar to subjects in others groups. Previous studies found subgroups of tinnitus patients with cluster analysis based on a small number of audiometric features^13^, a combination of features extracted from self-reports, audiometry and psychoacoustics^14^, a subset of socio-demographics, tinnitus characteristics, self-reports and audiological measurements^16^ or neuroimaging data and socio-demographics^17^. Although each of these studies provided insights in tinnitus subgroup patterns we believe that to increase acceptance amongst medical practitioners, clustering results need to be presented with intuitive visualisations that show individual subgroup patterns and enable the visual juxtaposition of multiple subgroups with respect to multi-variate data. With this in mind, Schlee et al. proposed a compact radar chart visualisation that allows to juxtapose the degree of health burden between either individuals or subgroups based on multi-variate data^18^. While their visualisation could be applied to any disease domain, Schlee et al. demonstrated its efficacy showing subgroup differences with respect to measurements of tinnitus distress and associated comorbidities. However, Schlee et al. did not aim to visualise clustering results, but restricted themselves to pre-defined cohorts such as female vs male patients or patients with low vs high tinnitus frequency.

Hence, the goal of this study is to combine clustering for tinnitus phenotyping with visual cluster representation and juxtaposition. Firstly, we performed cluster analysis and identified distinct phenotypes in patients with chronic tinnitus based on 64 (sub-)scales and sociodemographics extracted from self-report questionnaires. Secondly, we created an intuitive radial chart visualisation to display multi-variate characteristics of a single cluster, and to facilitate comparison of multiple clusters. Prospectively, the results of this study could be used for stratified research designs and treatment approaches.

## Materials and methods

### Patients

Analyses were based on data from N = 1228 patients with chronic subjective tinnitus who had been treated at the Tinnitus Center of Charité Universitätsmedizin Berlin, Germany, between January 2011 and October 2015. All patients had been suffering from tinnitus for 3 months or longer and were 18 years of age or older. Exclusion criteria comprised the presence of acute psychotic illnesses or addictions, deafness and insufficient knowledge of the German language. Multimodal psychosomatic assessments were carried out by ENT, internal, psychosomatic and physical therapy specialists. Treatment comprised an intensive, multimodal 7-day program that included informational counselling, detailed ear-nose-throat (ENT) as well as medical and psychological diagnostics, cognitive-behaviour therapy interventions, auditory training, relaxation exercises, and physiotherapy. Patients who presented with objective tinnitus were excluded from the present therapy and treated medically, as applicable. Ethical approval was granted by Charité Universitaetsmedizin Berlin ethics committee (reference number EA1/115/15). All relevant guidelines and regulations were followed. All patients gave informed written consent for data collection. Prior to the analyses, all data had been anonymised.

### Features

All patients completed a routine questionnaire assessment battery. These questionnaires were selected to obtain a comprehensive tinnitus assessment, including tinnitus-related distress and the psychosomatic background of tinnitus with anxiety, depression, general quality of life and experienced physical impairments. For clustering, a total of 64 features from 14 questionnaires was used, comprising 49 compound scores and 15 single-item measurements:

- The *Anamnestic Comparative Self-Assessment (ACSA)*^19^ is a visual analogue scale to measure the current quality of life (feature ACSA_qualityoflife).
- The *General Depression Scale (Allgemeine Depressionsskala; ADSL)*^20,21^ comprises 20 items for self-assessment of depressive symptoms including insecurity, exhaustion, hopelessness, self-devaluation, dejection, loneliness, sadness, lack of drive, perceived rejection by others, crying, enjoyment, withdrawal, fear, happiness, lack of reactivity, sleep disorders, appetite disorders, concentration problems and pessimism. Each item has 4 response options on an ordinal scale: 0 = “rarely or none of the time (less than 1 day)”, 1 = “some or a little of the time (1–2 days)”, 2 = “occasionally or a moderate amount of time (3–4 days)”, and 3 = “most or all of the time (5–7 days)”. We used the total sum score ADSL_depression.
- The *Berlin Complaint Inventory (Berliner Beschwerdeinventar; BI)*^22^ contains 57 items from the areas general well-being, autonomic nervous system, pain and emotionality. Two exemplary items are “I am bothered by feelings of weakness” and “I am bothered by nausea”. Respondents can answer on a 5-level ordinal scale with 0 = “not at all”, 1 = “hardly”, 2 = “somewhat”, 3 = “considerably” or 4 = “severely”. We used the 4 subscales exhaustion (BI_fatigue), abdominal symptoms (BI_abdominalsymptoms), limb pain (BI_limbpain), heart symptoms (BI_heartsymptoms) as well as the overall complaints sum score (BI_overallcomplaints).
- The *Berlin Mood Questionnaire (Berliner Stimmungsfragebogen; BSF)*^23^ constructs a multi-dimensional mood model from 30 items such as “I feel anxious” and “I feel belligerent” with 5 response options: 0 = “not at all”, 1 = “somewhat”, 2 = “rather”, 3 = “mainly” and 4 = “very much”. We used the following 6 subscales: fatigue (BSF_fatigue), apathy (BSF_apathy), anxious depression (BSF_anxdepression), anger (BSF_anger), positive mindset (BSF_mindset) and elevated mood (BSF_elevatedmood).
- The *ICD-10 Symptom Rating (ISR)*^24^ measures the severity of different mental disorders and comprises 36 items, including 29 single items on a 5-level ordinal scale: 0 = “does not apply”, 1 = “a little”, 2 = “quite a bit”, 3 = “to a great extent”, and 4 = “extremely”. We used the 6 subscales depressive syndrome (ISR_depression), anxiety syndrome (ISR_anxiety), obsessive–compulsive syndrome (ISR_compulsivesyn), somatoform syndrome (ISR_somatosyn), eating disorder syndrome (ISR_eatingdisorder), additional items score (ISR_additionalitems) and the total psychiatric syndrome score (ISR_totalpsychiatric-syn).
- The *(short form) Patient Health Questionnaire (PHQK)*^25^ measures the extent of depressive symptoms in the last 2 weeks and symptoms of anxiety in the last 4 weeks. The depressivity scale (PHQK_depression) is calculated as sum of 9 ordinal items (0 = “not at all”, 1 = “at some days”, 2 = “more than half of the days”, 3 = “almost every day”). The binary panic syndrome score (PHQK_panicsyn) is equal to “1” if all of the associated 5 “yes”/“no”-items are answered with “yes”.
- The *Perceived Stress Questionnaire (PSQ)*^26^ contains 20 items measuring subjective levels of stress within the last 4 weeks on a scale from 0 = “hardly ever” to 2 = “rarely”, 3 = “frequently” and 4 = “mostly”. We used the subscales demand (PSQ_demand), tension (PSQ_tension), joy (PSQ_joy), and worries (PSQ_worries) as well as the total perceived stress score (PSQ_stress).
- The *Pain Perception Scale (SES)*^27^ quantifies affective pain (SES_affectivepain) and sensoric pain (SES_sensoricpain) components as sum scores aggregated from a total of 24 items where the response options range from 1 = “does not apply at all” to 4 = “strongly applies”.
- *Visual Analogue Scales Pain (SSKAL)* consists of the 3 scales pain impairment (SSKAL_painimpairment), pain frequency (SSKAL_painfrequency) and pain intensity (SSKAL_painintensity).
- The *Short Form-8 Health Survey (SF8)*^28^ assesses 8 aspects of health-related quality of life. Each of the scores is composed of 8 items, each with a different set of 5 to 6 response options. We used the subscales bodily health (SF8_bodilyhealth), overall health (SF8_overallhealth), mental health (SF8_mentalhealth), physical functioning (SF8_physicalfunct), role emotional (SF8_roleemotional), role physical (SF8_rolephysical), social functioning (SF8_socialfunct), vitality (SF8_vitality), as well as the mental component summary score (SF8_mentalcomp) and the physical component summary score (SF8_physicalcomp).
- The *Self-Efficacy-Optimism-Pessimism Scale questionnaire (Selbstwirksamkeits-Optimismus-Pessimismus Skala; SWOP)*^29^ comprises 9 items on a scale from 1 = “not true” to 4 = “exactly true”. We used the scores on self-efficacy (SWOP_selfefficacy), optimism (SWOP_optimism) and pessimism (SWOP_pessimism).
- *Visual analogue scales (TINSKAL)* measuring tinnitus loudness (TINSKAL_loudness), frequency (TINSKAL_frequency) and distress (TINSKAL_distress) within a range between 0 and 10, respectively.
- From the *Tinnitus Localization and Quality Questionnaire (TLQ)*^30^, we extracted binary features indicating the location where the tinnitus is perceived as loudest (TINSKAL_01_leftear, TINSKAL_01_rightear, TINSKAL_01_bothears, TINSKAL_01_entirehead) and binary features on the sound that describe the tinnitus best (TINSKAL_02_whistling, TINSKAL_02_hissing, TINSKAL_02_ringing, TINSKAL_02_rustling).
- The German version of the *Tinnitus Questionnaire (TQ)*^31^ is an instrument to assess tinnitus-related distress and tinnitus severity. The questionnaire comprises 52 items with 3 levels each (0 = “true”, 1 = “partially true”, 2 = “not true”). We used the sum scores on auditory perceptual difficulties (TQ_auditoryperceptdiff), cognitive distress (TQ_cognitivedistress), emotional distress (TQ_emodistress), intrusiveness (TQ_intrusiveness), psychological distress (TQ_psychodistress), sleep disturbances (TQ_sleepdisturbances), somatic complaints (TQ_somaticcomplaints), as well as the total tinnitus distress score (TQ_distress).

Data from 2875 (70.1%) patients who did not complete all questionnaires were excluded due to missing values. Excluded patients were slightly, but significantly older than those included in the final sample ($\mu _{excluded}= 51.73$, $\sigma _{excluded}=13.63$; $\mu _{included}= 50.00$, $\sigma _{included}=11.91$; $t(2630.8)=-\,4.07, p<0.01$). Since all features of the SF8 and some features of the BSF, SWOP and PSQ have higher scores with better quality of life, features with a positive wording were reversed (new value = maximum feature value − old value) so that the interpretation (higher scores represent higher burden) remained consistent. Hereafter, feature names with a *-suffix denote reversed features. Due to widely differing value ranges, each feature was standardised prior to cluster analysis via z-score normalisation to have a mean of 0 and standard deviation of 1.

### Identification of tinnitus phenotypes using clustering

To identify a distinct set of tinnitus phenotypes, the clustering algorithm X-means was employed^32^. X-means is a parameter-free extension of the popular *K*-means clustering algorithm that incorporates the Bayesian Information Criterion^33^ (BIC) to automatically find an appropriate number of clusters *K* by finding a good trade-off between high goodness of fit and a low number of clusters. Let ${\mathscr {D}}$ be the dataset with *d* dimensions and *D* a subset of ${\mathscr {D}}$, i.e., $D\subseteq {\mathscr {D}}$. A *K*-means clustering on *D* yields the set of clusters ${\mathscr {C}}=\left\{ C_1,\ldots ,C_k,\ldots ,C_K\right\}$, where $c_k$ is the centroid of cluster *k*, $r_k$ is the number of points in *D* assigned to $c_k$ and *p* is the number of free parameters, i.e., $p = (d+1) \cdot K$. The BIC of a cluster $C_k$ using Schwarz criterion is calculated as $\text {BIC}(C_k) = {\hat{l}}_k({\mathscr {D}}) - \frac{p_k}{2} \cdot \log |{\mathscr {D}}|$, where ${\hat{l}}_k({\mathscr {D}})$ is the log-likelihood of ${\mathscr {D}}$ according to $C_k$. The point probabilities are computed as ${\hat{P}}(x_i)=\frac{r_{(i)}}{|{\mathscr {D}}|}\cdot \frac{1}{\sqrt{2\pi }{\hat{\sigma }}}\text {exp}\left( \frac{1}{2{\hat{\sigma }}^2} ||x_i-c_{(i)}||\right)$, where the maximum likelihood estimate for the variance (under the identical spherical Gaussian assumption) is ${\hat{\sigma }}^2=\frac{1}{|{\mathscr {D}}|-K}\sum _{i=1}^{|{\mathscr {D}}|}\left( x_i - \mu _{(i)}\right) ^2$. The log-likelihood of ${\mathscr {D}}$ according to ${\mathscr {C}}$ is $l({\mathscr {D}})=\log \prod _{i=1}^{|{\mathscr {D}}|} P(x_i)=\sum _{i=1}^{|{\mathscr {D}}|}\left( \log \frac{1}{\sqrt{2\pi }{\hat{\sigma }}} - \frac{1}{2 \sigma ^2} ||x_i-c_{(i)}||^2 + \log \frac{r_{(i)}}{|{\mathscr {D}}|} \right)$.

The X-means algorithm consists of 4 steps: (1) First, an initial K-means is run with $K=K_{lower}$. (2) Then each centroid is bisected into two children which are placed in opposite directions along a randomly chosen vector. (3) A “local” 2-means clustering is run for each pair of children and a BIC score is assigned to this new partitioning. along a randomly chosen vector. (4) If the BIC score increases with bisection of a centroid, the respective child centroids are kept, otherwise, the parent centroid is kept. Steps (2)–(4) are repeated until neither centroid’s BIC score can be improved by bisection. We used the R implementation of the X-means algorithm of Ishioka and set $K_{lower}$ to 2^34^. Numbering of clusters as cluster 1, cluster 2, etc. was done arbitrarily.

### Cluster visualisation

Visualisation of clusters in high dimensionality is challenging. The popular scatterplot matrices (SPLOMs) and their extensions intuitively represent the relation between pairs of features as matrix where each non-diagonal element is a two-dimensional scatterplot^35,36^. However, the number of scatterplots grows quadratically with increasing dimensionality which leads to scalability problems such as overplotting. Hence, advanced visualisation techniques have been proposed as a remedy, e.g. density contours, hexagon binning, coloring, transparency, layers showing aggregated geometric characteristics (minimal spanning trees, alpha shape, convex hull), animation, or combinations of multiple techniques such as splatterplots^37^. Still, SPLOMs and other traditional visualization techniques such as parallel coordinate charts^38^ are more suited for rather low-dimensional data with only a handful of features.

In case the original data cannot be adequately displayed on low-dimensional projections, dimensionality reduction (DR) is often applied as preprocessing step in advance of visualisation. DR algorithms transform a high-dimensional feature space onto a low-dimensional (often 2D) projection. Ideally, the projection preserves important structures of the original data, such as clusters, outliers, correlations and other important structures. Principal component analysis (PCA) is a frequently used DR algorithm that generates a new coordinate system with orthogonal dimensions^39^. The new dimensions (principal components, PC) are linear combinations of the original dimensions and are sorted according to variance. Each PC carries a loading that characterises how much variability of the data is explained. PCA is primarily suited for normally distributed data. However, PCA has problems with outliers and is incapable of capturing non-linear relationships. Another popular DR algorithm is multi-dimensional scaling (MDS) which puts emphasis on preserving distance. Points that are close in high-dimensional space should also be close in low-dimensional space. For complex (arbitrarily-shaped) structures, large distance is meaningless because of the curse of dimensionality, thus results may be unsatisfactory. *t*-stochastic neighbourhood embedding (*t*-SNE) is a non-linear dimensionality reduction technique that visualises a matrix of pairwise-similarities^40^. The similarities are calculated in a way to both preserve global structures (clusters at different scales) and local structures (distances and neighbours). *t*-SNE does not allow for interpretation of the original dimensions. Further, the technique does not support to add a new observation to the existing projection without recalculation.

DR techniques cannot be applied here because even if the clustering structure is preserved in the data projection, the semantics of the original features will be lost. Discussions with domain experts led to the following requirements for a cluster visualisation:

- preservation of original features,
- intuitive cluster representation for multi-variate data with dozens of features,
- compact, at a glance comparison of multiple clusters,
- chart design that allows to juxtapose a cluster with the overall patient mean.

We therefore introduce a new radial bar chart visualisation as graphical representation of a single cluster, enriched with dedicated elements that satisfy the aforementioned requirements. In particular, the height of a bar depicts the average value of a feature over the patients assigned to that cluster. The radial spatial layout distributes the bars around a circle where each bar starts at the black 0 line which represents the feature average over all patients included in this study. Due to the scaling (z-score normalisation) of the features, bars inclined to the outside represent feature averages above the overall patient mean and bars inclined to the inside represent feature averages below the overall patient mean. This interpretation is visually supported by colour-coded bars using a sequential gradient from dark blue (low burden) to yellow (mean burden) to bright red (high burden). Feature names are shown on top of each bar. All values are depicted in terms of standard deviation away from the mean. For example, a value of − 1 indicates that the cluster average is 1 standard deviation smaller than the overall patient average. Intra-cluster standard deviation are represented as grey error lines facing the coloured inner circle. To facilitate quick feature localisation, features were grouped into categories which are displayed in the inner circle, alongside the cluster name and the number of patients assigned to that cluster. These categories were (in clockwise order): tinnitus characteristics, physical quality of life, experiences of pain, somatic expressions, affective symptoms, tinnitus-related distress, internal resources, perceived stress, and mental quality of life.

To provide a graphical overview of all clusters at the same time, we designed a radar chart variant where feature averages are represented as points instead of bars which allows to show multiple clusters. Within each feature category, the points of a cluster are connected by line segments. Points and line segments are coloured by cluster.

#### Interactive components for cluster inspection

To provide a graphical overview, an interactive demo of the cluster solutions and the visualisations is available under https://unmnn.de/phs/app/. Radar charts were augmented with interactive components: by hovering over a bar or a feature label, additional cluster summaries and compact feature descriptions are shown as tooltips. Clicking on a feature invokes an additional chart which shows the (normalised) distribution of the selected feature stratified by cluster, and if selected, also after treatment. Continuous features are shown using semi-transparent boxplots placed on violin plot^41^ layers whereas for nominal features, category proportions alongside their 95% confidence intervals are displayed as points and error lines, respectively.

### Statistical methods

Kruskal–Wallis test was used for statistical comparison of inter-phenotype differences for continuous features (like age), and Pearson’s chi-squared test was used for categorical features (like gender). Differences were considered significant if $p<0.05$. Correction for multiple comparison was not conducted due to the exploratory nature of the study. Confidence intervals for the means were estimated using nonparametric basic bootstrap sampling^42^ with 2000 samples, respectively.

## Results

**Figure 1:**
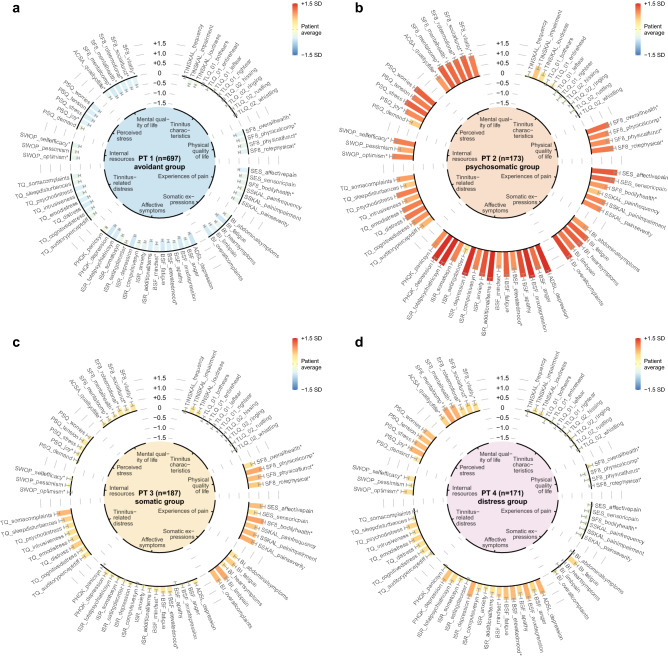
Radial barcharts visualizing the 4 phenotypes. (**a**) Phenotype 1 (PT1) characterises the patient subgroup with lowest health burden among all phenotypes. (**b**) PT2 represents the most suffering subgroup, with all of the psychosomatic and somatic measurement averages exceeding the population mean $+$ 0.5 standard deviations (SD). PT3 (**c**) exhibits above population average scores for somatic indicators whereas PT4 (**d**) is characterised by increased distress scores, including subjective stress and perceived quality of life. Bars are arranged in a circular layout. The height of a bar shows a feature’s z-score normalised within-cluster average, and the grey line centred at the top of the bar illustrates the 95% confidence interval. The colour of a bar represents the difference of the within-cluster average from the overall patient average (PA), from − 1.5 SD below PA (dark blue) to PA (yellow) and $+$ 1.5 SD above PA (bright red). Features were grouped into 9 categories defined by tinnitus experts. The categories are shown within the inner circle. See subsection Features for a description of each questionnaire and the extracted features.

**Figure 2:**
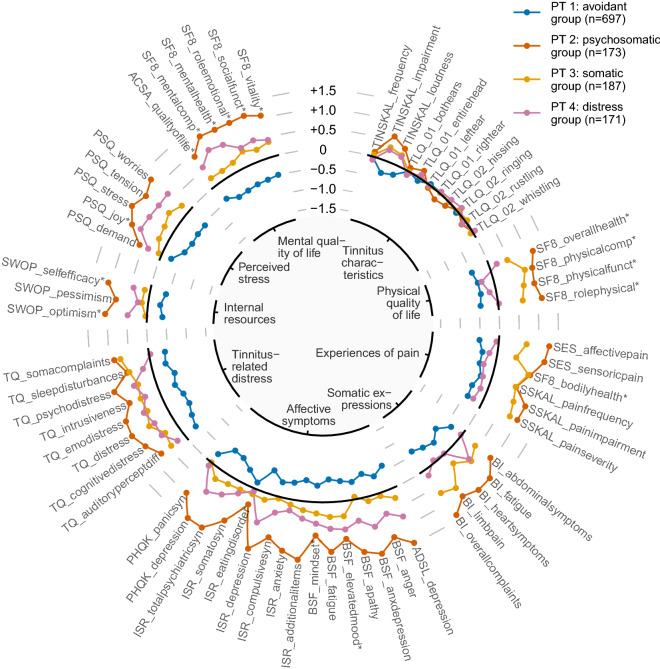
Radial line chart juxtaposing the 4 phenotypes. In the chart, a point shows a feature’s (z-score normalised) within-phenotype average. In each feature category (labels in inner circle), points are connected with line segments. Points and lines are coloured by cluster.

According to *X*-means, four clusters (referred to as phenotypes hereafter) represent an optimal solution for the given dataset. The radial barcharts in Fig. 1 visualise phenotype-individual averages for all features. Graphical summaries of phenotype value distributions for all features on their original scales are provided in Supplementary Fig. S1. The radar plot in Fig. 2 shows average scores for each variable and for all clusters. While the four phenotypes are clearly distinguishable with respect to the psychosomatic and somatic variables, the line segments for most features of the group “tinnitus characteristics” are close to the overall patient average. Phenotype 1 (PT 1) represents the largest subgroup with 697 out of 1,228 patients (56.8%). This patient subgroup is characterised by ostensibly below-average symptom expression across tinnitus-related and broader psychosomatic symptom indices, including affective symptoms, perceived stress, tinnitus-related distress and somatic symptoms, as well as (above-average) quality of life and internal resources (Fig. 1). Due to their help-seeking behaviour, presentation in clinic and wish to participate in multimodal treatment, it can be assumed that this group of patients do experience psychological distress, however aim to present themselves as healthily as possible. We therefore label this phenotype “avoidant group”. Patients in this subgroup feature proportionately high levels of education, employment and low levels of leave of absence and psychotherapeutic treatment (Table 1). PT 2 comprised 173 patients (14.1%) who reported the highest emotional and somatic burden among all PTs (Fig. 1b). More specifically, PT 2 represents a patient subgroup with high psychosomatic-comorbidity and is thus labelled “psychosomatic group”. This patient subgroup shows high tinnitus burden alongside clinically relevant impairment across all affective indices including depression, anxiety, and perceived stress. These affective symptoms appear to align with somatoform expressions of distress including somatic symptoms. Patients of these subgroup report severely reduced quality of life and reduced coping opportunities with more pessimism, less experienced self-efficacy and optimism. Patients in this subgroup feature a high proportion of patients who live alone, are unemployed or show an overall lower educational status. Patients in this cluster further appear to consult more doctors, take more leave of absence and use more psychotherapy. PT 2 patients reported the tinnitus sound to be audible in the entire head to a greater portion than the other groups. PT 3 contained 187 associated patients (15.2%) characterised by above-average scores of features measuring somatic complaints and near-average scores for affective symptoms (Fig. 1c). Since pain scores of SF8_bodilyhealth* and SSKAL_painfrequency were similarly large as PT2, this patient subset was labelled “somatic group”. PT 3 represented the oldest subgroup, with the largest proportion of female patients and largest reported time period since tinnitus onset. Unlike PT3, PT 4 (n = 171; 13.9%) exhibited above-average scores for affective scores, components of quality of life and perceived stress (Fig. 1d), e.g. mental component summary score (SF8_mentalcomp*; 0.85) and anxious depression score (BSF_anxdepression; 0.79). Hence, we label PT 4 as “distress group”. PT 4 comprises the youngest of the 4 subgroups, with the largest fraction of male patients (Table 1).

**Table 1:**
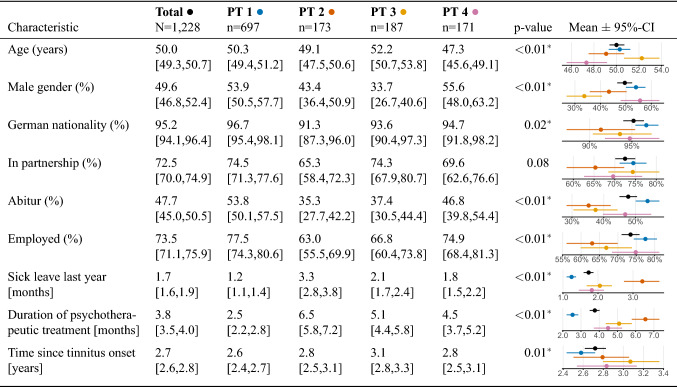
Inter-group comparison of socio-demographics. Summaries of socio-demographic features are given as means [95% confidence interval] for all patients and for each of the 4 phenotypes. An asterisk indicates whether ${p}<0.05$.

## Discussion

In this study, we combined data-driven clustering with a novel visualisation to identify and display distinct phenotypes in a large sample of patients with chronic tinnitus. Patient data were extracted from self-report questionnaires prior to starting a multimodal treatment program. Our analysis suggests four phenotypes of patients with chronic tinnitus.

PT 1 (*avoidant group*) represents a large proportion of patients. Apart from the tinnitus symptom, patients in this subgroup reported few other affective or psychosomatic symptoms, and the tinnitus is used as an index- representation of experienced distress. Due to these patients’ focused presentation (“*everything is okay were it not for the tinnitus*”), clinicians can easily be led to believe that potential other contributors to individual distress must not require assessment. However, clinical experience strongly suggests that a thorough assessment of broader psychosocial stressors is warranted in so far as it is feasible in clinical practice environments. The psychosocial resourcefulness of this subgroup enables patients to seek help quickly and in a solution-focused manner. Good tinnitus-specific counselling and individualised (online) therapy modules featuring audiological, psychological or relaxation procedures would possibly represent an adequate treatment strategy for this patient subgroup.

PT 2 (*psychosomatic group*) represents 15% of patients who showed high tinnitus burden alongside clinically relevant impairment across all affective indices including depression, anxiety, and perceived stress. These affective symptoms appear to align with somatoform expressions of distress including physical complaints and somatic symptoms. Patients of these subgroup report severely reduced quality of life and reduced coping opportunities with more pessimism, less experienced self-efficacy and optimism. There is the frequently asked question as to whether increased tinnitus-related distress contributes to increases in depression or vice versa. In this group, we consider depressive or anxious symptoms to be a crucial underlying factor for general symptom burden and treatment must begin with a focus on improving mood and relieving depression. Here, tinnitus-related distress needs to be seen within a broader context of medical and psychological influencing factors that require idiosyncratic conceptualisation. According to the socio-demographic variables, this patient subgroup features a higher proportion of women, and more patients who live alone, are unemployed or show an overall lower educational status. Patients in this cluster further appear to consult more doctors, take more leaves of absence and use more psychotherapy.

PT 3 (*somatic group*) appears to represent a patient subgroup that is characterised by somatopsychic symptom expressions, i.e., physical symptoms that may reflect distress and/or underlying medical conditions. To adequately address the needs of this patient subgroup, multimodal interventions might include a proportion of body-oriented procedures such as relaxation exercises or physiotherapy whose effect, however, should be interpreted with regard to both direct and indirect psychological effects (e.g. through increased senses of well-being or others’ care).

Patients in PT 4 (*distress group*) reported above-average perceived stress, accompanied with physical exhaustion and anxious-depressive mood. This group includes rather younger, employed patients (more men), who indicated chronic distress, potentially being susceptible to burnout syndrome with subjective reduced mental capacity (“hamster wheel”), which is used as a description of the life situation even without tinnitus stress. In this subgroup, tinnitus might represent chronic stress associated with psychological vulnerabilities, environmental/work stressors and dysfunctional coping strategies. Multimodal therapy should initially focus on stress-regulation techniques, including relaxation or individually tailored behavioural modification approaches. Similar to the highly psychosomatically burdened PT 2, patients in PT 4 could also benefit from longer psychotherapeutic or multimodal treatment procedures (inpatient or rehabilitative).

The overview and juxtaposition of all clusters shows that some questionnaires and characteristics contribute a lot to differences among patient phenotypes. In particular, patient phenotypes differ substantially with respect to their coping attitudes, their stress and their perception of quality of life, as well as their tinnitus distress. Some of the questionnaire items separate well among some of the phenotypes, see e.g. the items on perceived pain and complaints. In contrast, patients do not seem to differ in their perception of tinnitus. These contributions of the questionnaires to the phenotypes indicate that phenotyping may be achievable also with less questionnaires, especially because some of the questionnaires are overlapping.

Previous studies also employed clustering algorithms to identify tinnitus subtypes^13,14,16,17^. It is difficult to compare our findings to theirs because of the different set of available measurements: whereas the strength of our study was a large pool of self-report questionnaire data, Tyler et al. used both self-report data and audiometrics^14^, Schecklmann et al. used self-report data and cordiac imaging features^17^, and Langguth et al. used audiometric data^13^. Nevertheless, PT 2 (psychosomatic suffering group) appears to match the “constant distressing tinnitus” subgroup reported by Tyler et al.^14^, as average scores on features measuring tinnitus-related health burden were distinctly greater than in the other subgroups. Of course, the selection of meaningful features is pivotal for the efficacy of any cluster analysis. Schlee et al. argued against the usage of single-item features like visual analogue scale measurements because of their higher susceptibility to random measurement errors, lower test-retest reliability and higher vulnerability to unknown biases^18^. Due to the exploratory nature of our study, we decided to include both single-item measurements and compound scores. We assigned 11 out of 15 single-item measurements into the category “Tinnitus characteristics”. Figure 2 (top-right) shows their low discriminative power, as all phenotypes’ means are close to the population average, with the exception of TINSKAL_impairment and TINSKAL_loudness. Future research might focus on identifying a subset of key questionnaires alongside a simple computational tool that will enable clinicians to match individual patients with one or more of the here identified phenotypes.

Closest to our radial bar chart visualisation is the radar chart proposed by Schlee et al.^18^. Their solution indeed facilitates the comparison of two subgroups by comparing the areas of their associated polygons. However, there is still the potential problem of overplotting when one wishes to compare more than 2 subgroups as we do. Hence, we did not opt to fill up the areas spanned by the connected points with colour, to avoid a polygonal that fully overlays another one. Further, since the main criterion for comparison is the polygonals’ shapes, inferences highly depend on the ordering of features around the plot which can be misleading. Schlee et al.^18^ tackled this problem by computing an ordering that yields areas that achieve maximum mean surface difference between subgroups and minimum surface variance within subgroups. This approach is feasible for up to a moderate ($\approx \,20$) number of features. In our study with 64 features, we decided to arrange features based on semantic categories, e.g. quality of life. This allows to detect and track features easier. Further, our visualisation is not specific to tinnitus but could be used to present a compact visual summary of characteristics of any condition or index symptoms subgroups. Whether the visualisations will be adopted by clinicians for finding suitable tinnitus management strategies needs to be tested. Preliminarily, clinicians suggested that graphical summaries of possible patient subtypes may alleviate allocation of modular treatment strategies to specific combinations of symptom presentations.

A potential limitation of our analysis is the exclusion of patients who did not fill all questionnaires during admission. There are several reasons why a patient may not have filled all questionnaires, including unfamiliarity with the technical devices (the questionnaires must be filled electronically), loss of motivation due to the relatively large number of questionnaires and collision with baseline examinations in the lab. The exclusion of these patients may have led to a selection bias. Nonetheless, our analysis over all 15 questionnaires allowed us to acquire insights to the contribution of these questionnaires to phenotyping, so that eventually a reduction of questionnaires might become possible. Further, our analysis is a static snapshot of phenotypes at baseline. Hence, it might be sensitive to possible changes in tinnitus perception and associated health burden over time. It is possible that a patient will transition from one phenotype to another in later stages of her life or depending on her tinnitus management. Thus, a next step would be to study the effects of treatment to these phenotypes and find whether some patient phenotypes benefit more than others. Another limitation comes from the heuristic choice of the number of phenotypes. Many works use the number of clusters as input parameter. Since this number is not known, we used the non-parametric *X*-Means clustering algorithm.

## Supplementary information

Supplementary information.

## Data Availability

The datasets for this article are not publicly available because no consent of the patients to publish their data was obtained. Notwithstanding, interested researchers can contact the directorate of the Tinnitus Center Charité Universitaetsmedizin Berlin with data access requests addressed at the senior author [birgit.mazurek@charite.de].
