# Supplementary material for: Phenotyping chronic tinnitus patients using self-report questionnaire data: cluster analysis and visual comparison
Source: Sci Rep. 2020 Oct 2;10:16411. doi: 10.1038/s41598-020-73402-8 (PMC7532444; doi:10.1038/s41598-020-73402-8)
Supplement: Supplementary file 1 — Supplementary information. [file 41598_2020_73402_MOESM1_ESM.pdf]

–Supplementary Material–
Phenotyping chronic tinnitus patients using self-report questionnaire data: cluster analysis and visual comparison

Uli Niemann[1,*], Petra Brueggemann[2], Benjamin Boecking[2], Wilhelm Mebus[2], Matthias Rose[3], Myra Spiliopoulou[1], and Birgit Mazurek[2]

[1] *Faculty of Computer Science, Otto von Guericke University Magdeburg, Magdeburg, Germany*
[2] *Tinnitus Center, Charité Universitaetsmedizin Berlin, Berlin, Germany*
[3] *Division of Psychosomatic Medicine, Medical Department, Charité Universitaetsmedizin Berlin, Berlin, Germany*
[*] *Corresponding author: uli.niemann@ovgu.de*

Supplementary Figure S1

Graphical summary of feature value distribution for each phenotype. For numerical features, semi-transparent boxplots are shown on top of dashed violin plots. A diamond symbol depicts the mean value. Barcharts are shown for proportion features. Features are presented in the same order as in Figure 1 (clockwise).

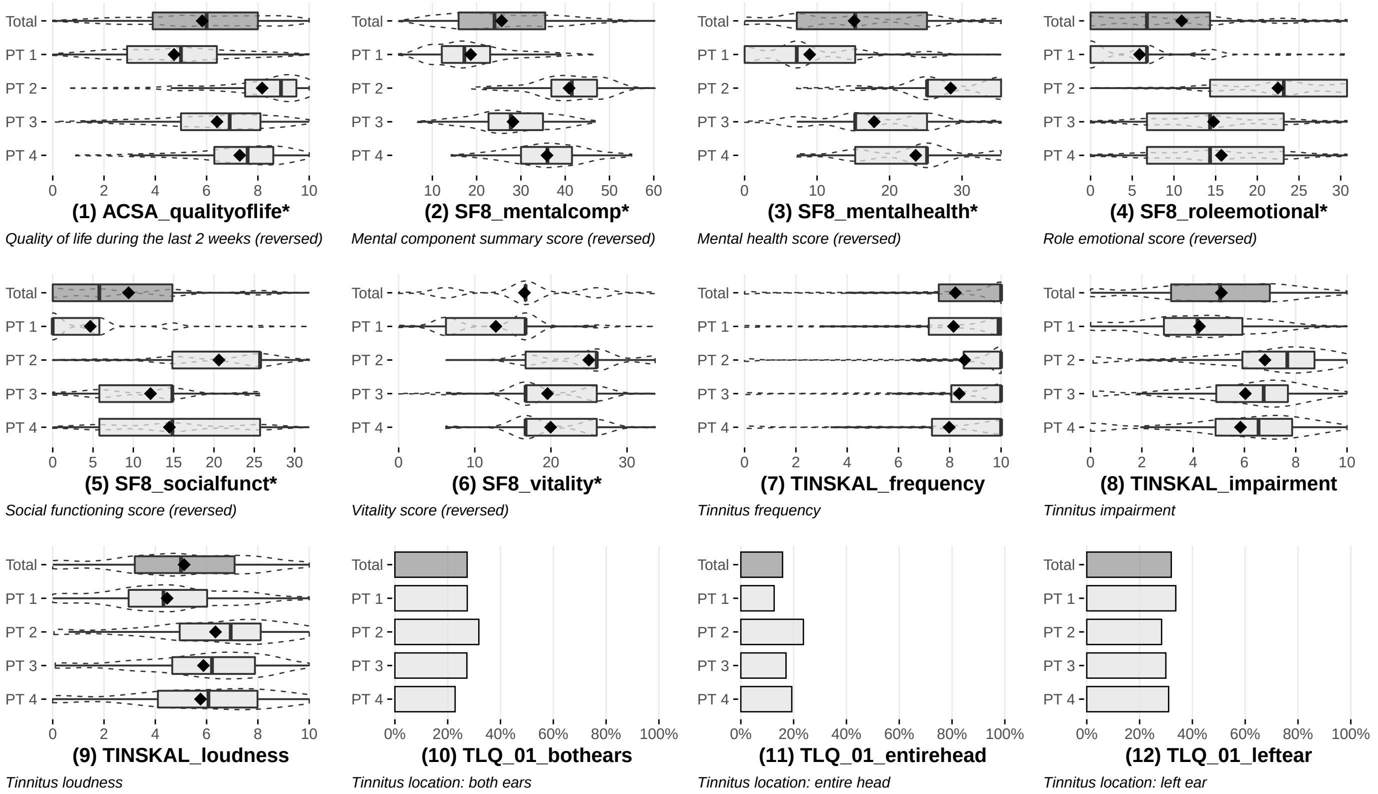

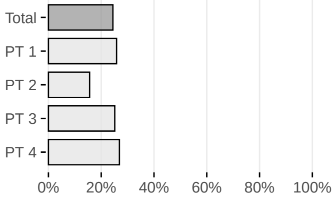

(13) TLQ_01_rightear

Tinnitus location: right ear

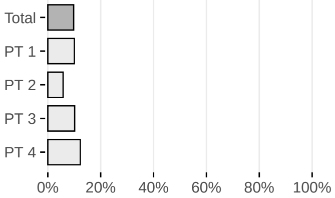

(14) TLQ_02_hissing

Tinnitus noise: hissing

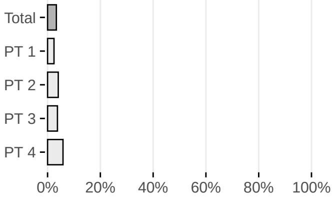

(15) TLQ_02_ringing

Tinnitus noise: ringing

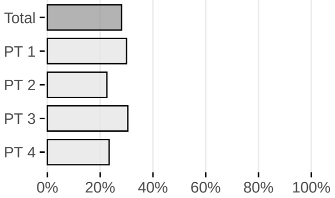

(16) TLQ_02_rustling

Tinnitus noise: rustling

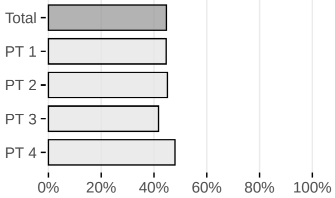

(17) TLQ_02_whistling

Tinnitus noise: whistling

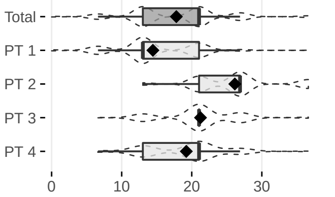

(18) SF8_overallhealth*

Overall health score (reversed)

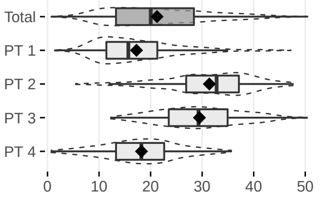

(19) SF8_physicalcomp*

Physical component summary score (reversed)

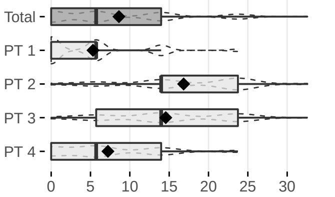

(20) SF8_physicalfunct*

Physical functioning score (reversed)

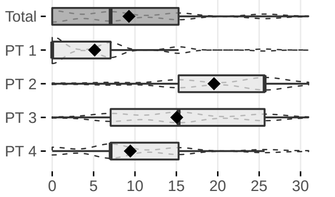

(21) SF8_rolephysical*

Role physical score (reversed)

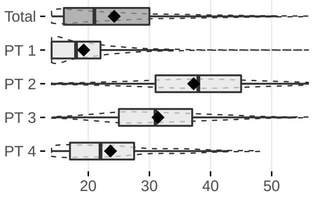

(22) SES_affectivepain

Affective pain

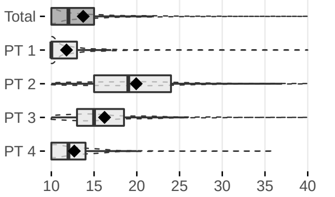

(23) SES_sensoricpain

Sensoric pain

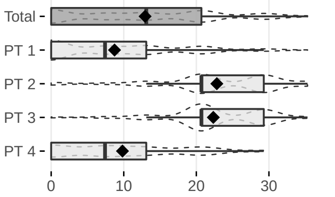

(24) SF8_bodilyhealth*

Bodily health score (reversed)

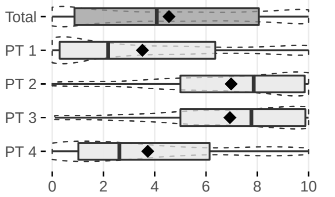

(25) SSKAL_painfrequency

Visual analog scale pain frequency

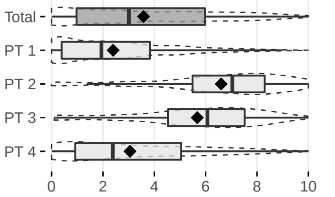

(26) SSKAL_painimpairment

Visual analog scale pain impairment

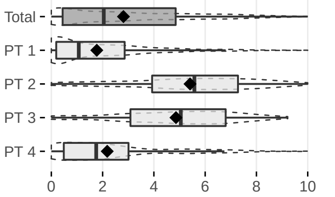

(27) SSKAL_painseverity

Visual analog scale pain severity

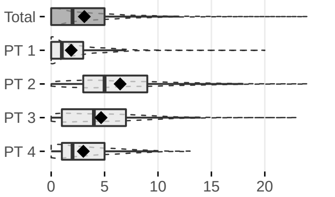

(28) BI_abdominalsymptoms

Abdominal symptoms score

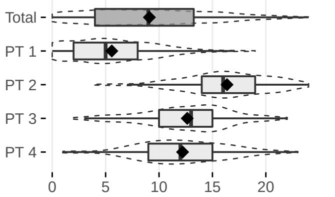

(29) BI_fatigue

Fatigue score

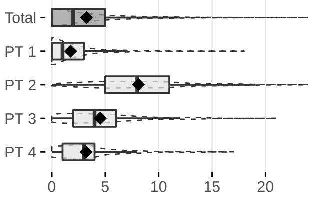

(30) BI_heartsymptoms

Heart symptoms score

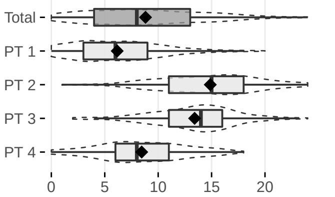

(31) BI_limbpain

Limb pain score

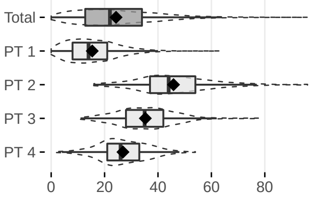

(32) BI_overallcomplaints

Overall complaints sum score

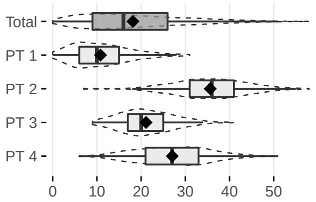

(33) ADSL_depression

Depressive disorder sum score

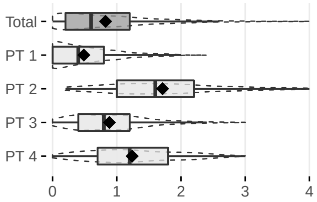

(34) BSF_anger

Anger score

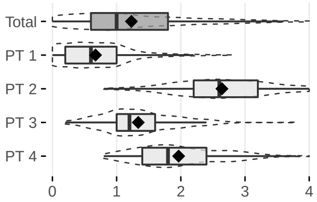

(35) BSF_anxdepression

Anxious depression score

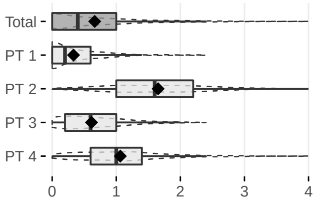

(36) BSF_apathy

Apathy score

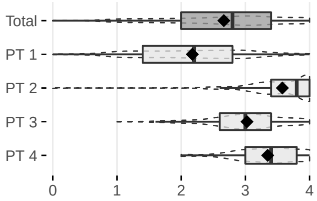

(37) BSF_elevatedmood*

Elevated mood score (reversed)

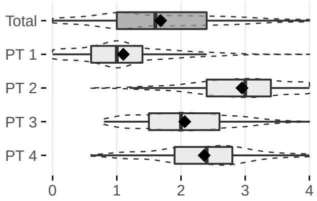

(38) BSF_fatigue

Fatigue score

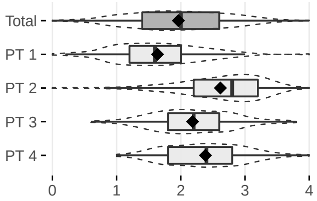

(39) BSF_mindset*

Positive mindset score (reversed)

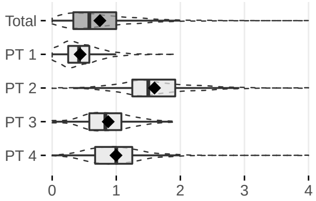

(40) ISR_additionalitems

Additional items score

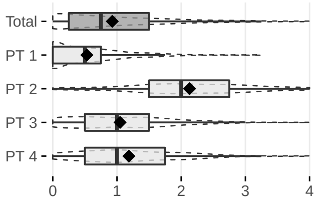

(41) ISR_anxiety

Anxiety score

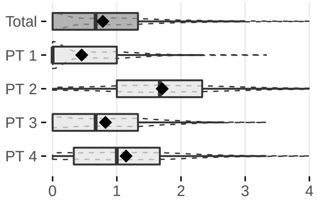

(42) ISR_compulsivesyn

Obsessive−compulsive syndrome score

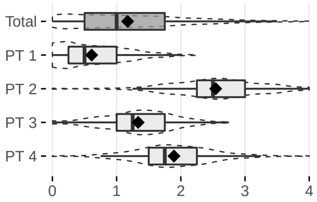

(43) ISR_depression

Depression score

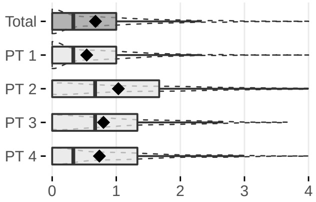

(44) ISR_eatingdisorder

Eating disorder score

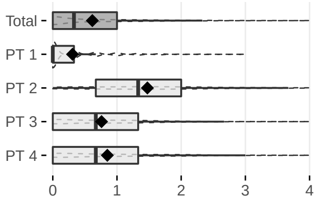

(45) ISR_somatosyn

Somatoform syndrome score

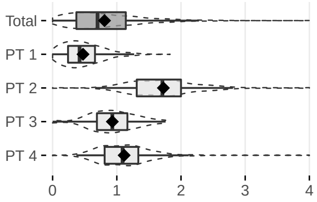

(46) ISR_totalpsychiatricsyn

Total psychiatric syndrom score

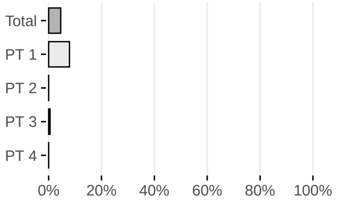

(47) PHQK_depression

Presence of depression

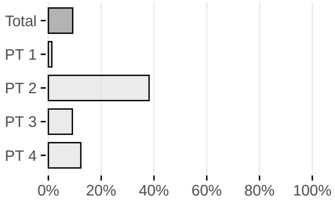

(48) PHQK_panicsyn

Presence of panic syndrome

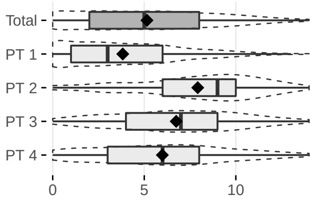

(49) TQ_auditoryperceptdiff

Auditory perceptual difficulties score

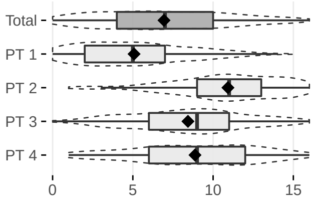

(50) TQ_cognitivedistress

Cognitive distress score

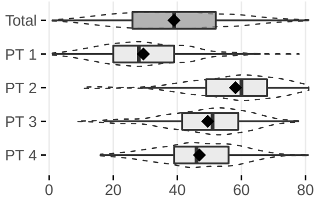

(51) TQ_distress

Total tinnitus distress score

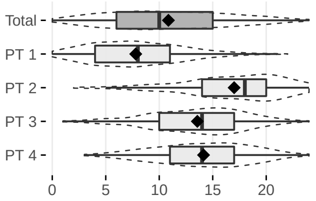

(52) TQ_emodistress

Emotional distress score

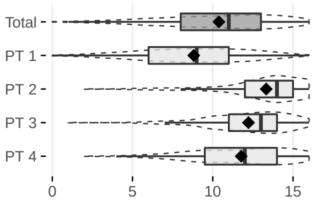

(53) TQ_intrusiveness
Intrusiveness score

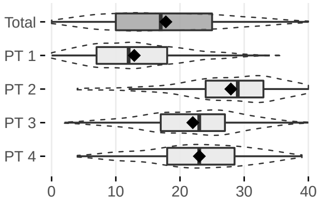

(54) TQ_psychodistress
Psychological distress score

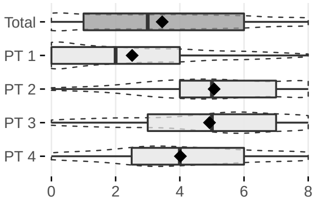

(55) TQ_sleepdisturbances
Sleep disturbances score

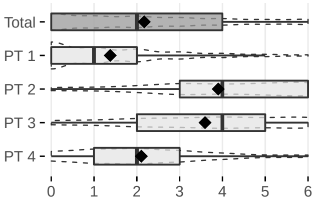

(56) TQ_somacomplaints
Somatic complaints score

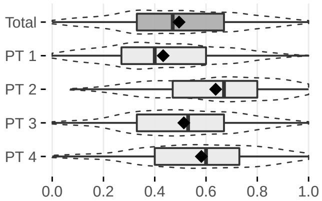

(57) PSQ_demand
Demand score

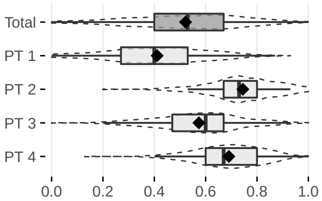

(58) PSQ_joy*
Joy score (reversed)

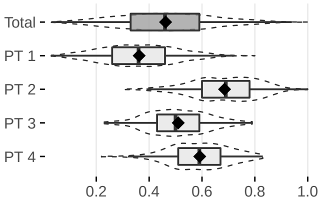

(59) PSQ_stress
Total perceived stress sum score

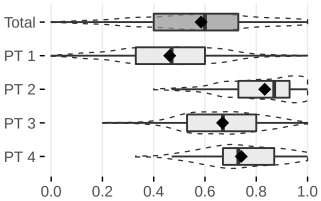

(60) PSQ_tension
Tension score

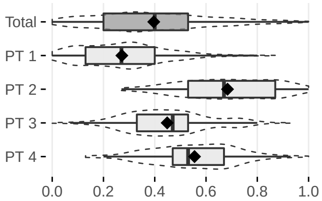

(61) PSQ_worries
Worries score

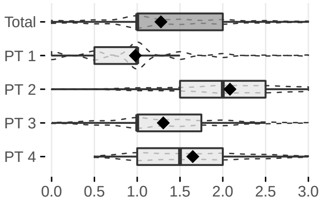

(62) SWOP_optimism*
Optimism score (reversed)

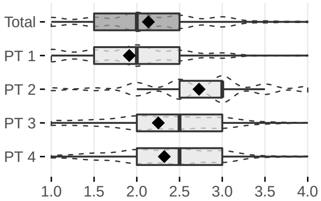

(63) SWOP_pessimism
Pessimism score

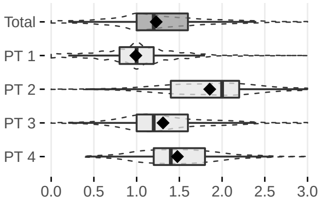

(64) SWOP_selfefficacy*
Self−efficacy score (reversed)
